# Supplementary material for: Acceptability of pre-exposure prophylaxis and associated factors among HIV-negative young men in Kagwara fishing community-Serere district, Uganda: A cross-sectional study
Source: PLoS One. 2025 Jun 4;20(6):e0317808. doi: 10.1371/journal.pone.0317808 (PMC12136446; doi:10.1371/journal.pone.0317808)
Supplement: S2 File — (PDF) [file pone.0317808.s002.pdf]

**06/04/2023**

To: alex omoding

busitema university
+256782464769

**Type:** Initial Review

**Re: BUFHS-2023-61: ACCEPTABILITY OF HIV PRE-EXPOSURE PROPHYLAXIS AND ITS ASSOCIATED FACTORS AMONG HIGH-RISK HIV-NEGATIVE YOUNG MEN IN KAGWARA FISHING COMMUNITY – SERERE DISTRICT, ,**

I am pleased to inform you that at the **10** convened meeting on **06/04/2023**, the Busitema University Faculty of Health Sciences REC, committee meeting, etc voted to approve the above referenced application.
Approval of the research is for the period of **06/04/2023** to **06/04/2024.**

As Principal Investigator of the research, you are responsible for fulfilling the following requirements of approval:

1. All co-investigators must be kept informed of the status of the research.
2. Changes, amendments, and addenda to the protocol or the consent form must be submitted to the REC for re-review and approval **prior** to the activation of the changes.
3. Reports of unanticipated problems involving risks to participants or any new information which could change the risk benefit: ratio must be submitted to the REC.
4. Only approved consent forms are to be used in the enrollment of participants. All consent forms signed by participants and/or witnesses should be retained on file. The REC may conduct audits of all study records, and consent documentation may be part of such audits.
5. Continuing review application must be submitted to the REC **eight weeks** prior to the expiration date of **06/04/2024** in order to continue the study beyond the approved period. Failure to submit a continuing review application in a timely fashion may result in suspension or termination of the study.
6. The REC application number assigned to the research should be cited in any correspondence with the REC of record.
7. You are required to register the research protocol with the Uganda National Council for Science and Technology (UNCST) for final clearance to undertake the study in Uganda.

The following is the list of all documents approved in this application by Busitema University Faculty of Health Sciences REC:

| No. | Document Title | Language | Version Number | Version Date |
|---|---|---|---|---|
| 1 | Data collection tools | ATESO | Version 2 | 2023-02-26 |
| 2 | Data collection tools | English | Version 2 | 2023-02-26 |
| 3 | Assent form if applicable to your study | ATESO | version 1 | 2023-02-26 |
| 4 | Assent form if applicable to your study | English | version 1 | 2023-02-27 |
| 5 | clean protocol | English | Version 2 | 2023-02-27 |
