# Supplementary material for: Acceptability of pre-exposure prophylaxis and associated factors among HIV-negative young men in Kagwara fishing community-Serere district, Uganda: A cross-sectional study
Source: PLoS One. 2025 Jun 4;20(6):e0317808. doi: 10.1371/journal.pone.0317808 (PMC12136446; doi:10.1371/journal.pone.0317808)
Supplement: Attachment — Submitted filename: PONE-D-24-60531 MANUSCRIPT HAVING REVIEW COMMENTS.pdf [file pone.0317808.s004.pdf]

PLOS ONE

Acceptability of pre-exposure prophylaxis and associated factors among HIV-negative young men in Kagwara fishing community- Serere district: a cross-sectional study

--Manuscript Draft--

**Manuscript Number:**	PONE-D-24-60531
**Article Type:**	Research Article
**Full Title:**	Acceptability of pre-exposure prophylaxis and associated factors among HIV-negative young men in Kagwara fishing community- Serere district: a cross-sectional study
**Short Title:**	Short Title: PrEP acceptability among HIV negative young men.
**Corresponding Author:**	Ronald Opito, MPH<br>Soroti University<br>Soroti, UGANDA
**Keywords:**	HIV Pre-Exposure Prophylaxis, acceptability, factors, young men, fishing community.
**Abstract:**	Background: Despite the potential efficacy of Pre-Exposure Prophylaxis (PrEP) in reducing HIV risk among at-risk populations, PrEP acceptability remains strikingly varied across populations. We assessed PrEP acceptability and associated factors among at-risk HIV-negative young men in Kagwara fishing community, Serere district.<br>Methods: A cross-sectional quantitative study design was used. Data were collected among 409 at-risk HIV-negative young men aged 15-24 years living in Kagwara fishing community, between August and October 2023. Quantitative data were collected on socio-demographic characteristics, sexual-risk behaviors, knowledge, attitudes, and practices regarding PrEP acceptability. PrEP acceptability was defined as the proportion of young men accepting to use PrEP out of those interviewed, based on six constructs adopted from acceptability framework. Data analyzed using Stata version 15.0 statistical software. Summary statistics computed and presented as tables, frequencies and proportions. Bivariate analysis was conducted using penalized logistic regression to identify independent factors associated with PrEP acceptability. All factors that had p<0.10 at the bivariate analysis and suspected confounders were entered into the final logistic regression model. All factors with p<0.05 were significantly associated with the primary outcome.<br>Results: Of 409 respondents, average age was 21.8(SD=1.9) years. Majority, (97.8%, n=393) had unprotected penetrative sex while 84.6% (n=346) did not know HIV status of their partners. PrEP acceptability was high at 93.6% (n=383) as majority of the participants accepted to use PrEP based on the six constructs of acceptability. At multivariable level, the factors associated with PrEP acceptability were; perceived risk of getting HIV infection, adjusted odds ratio (aOR)(95%CI)=4.23(1.05, 17.04), knowing the partner's HIV status, aOR (95%CI) = 0.25 (0.07, 0.88), feeling embarrassed to ask for PrEP from the facility, aOR (95%CI) = 0.12 (0.04, 0.39), and having concern of stigma associated with use of PrEP, aOR = 0.13 (95% CI, 0.04-0.41).<br>Conclusion: We found a high level of PrEP acceptability among at-risk HIV-negative young men in Kagwara fishing community. Improving access to PrEP services among at-risk young men in the fishing communities may increase PrEP uptake in this population and across similar settings.
**Order of Authors:**	Alex Omoding, BNS, MPH
	Ronald Opito, MPH
	Paul Oboth, MSc
	Francis Okello, MSc QE
	Joseph KB Matovu, PhD
**Additional Information:**	
**Question**	**Response**
**Financial Disclosure**	The author(s) received no specific funding for this work.

Powered by Editorial Manager® and ProduXion Manager® from Aries Systems Corporation

Enter a financial disclosure statement that describes the sources of funding for the work included in this submission. Review the submission guidelines for detailed requirements. View published research articles from *PLOS ONE* for specific examples.<br><br>This statement is required for submission and **will appear in the published article** if the submission is accepted. Please make sure it is accurate.<br><br>**Funded studies**<br>Enter a statement with the following details:<br>• Initials of the authors who received each award<br>• Grant numbers awarded to each author<br>• The full name of each funder<br>• URL of each funder website<br>• Did the sponsors or funders play any role in the study design, data collection and analysis, decision to publish, or preparation of the manuscript?<br><br>Did you receive funding for this work?	
**Competing Interests**<br><br>Use the instructions below to enter a competing interest statement for this submission. On behalf of all authors, disclose any competing interests that could be perceived to bias this work—acknowledging all financial support and any other relevant financial or non-financial competing interests.<br><br>This statement is required for submission and **will appear in the published article** if the submission is accepted. Please make sure it is accurate and that any funding sources listed in your Funding Information later in the submission form are also declared in your Financial Disclosure statement.	The authors have declared that no competing interests exist.

View published research articles from *PLOS ONE* for specific examples.<br><br>**NO authors have competing interests**<br><br>Enter: *The authors have declared that no competing interests exist.*<br><br>**Authors with competing interests**<br><br>Enter competing interest details beginning with this statement:<br><br>*I have read the journal's policy and the authors of this manuscript have the following competing interests: [insert competing interests here]*<br><br>* typeset	
**Ethics Statement**<br><br>Enter an ethics statement for this submission. This statement is required if the study involved:<br><br>• Human participants<br>• Human specimens or tissue<br>• Vertebrate animals or cephalopods<br>• Vertebrate embryos or tissues<br>• Field research<br><br>Write "N/A" if the submission does not require an ethics statement.<br><br>General guidance is provided below. Consult the submission guidelines for detailed instructions. **Make sure that all information entered here is included in the Methods section of the manuscript.**	Ethical approval to conduct the study was obtained from Busitema University Research and Ethics Committee (REC), Approval number BUFHS-2023-61. Administrative clearance was obtained from the district health office, Serere district and the local government representative office in whose jurisdiction Kagwara landing site was situated. Given that the study was assessing behavior related to HIV prevention among high-risk HIV- negative persons, this certainly needed to uphold several ethics when conducting the study. Each participant at his own discretion provided an informed consent (written) as was indicated on the consent form, while parental consent was obtained for all the minors prior to their participation in the study. Confidentiality was maintained by ensuring that individual patient-level data obtained was de-identified, encrypted, and passworded to ensure access by only an authorized team of investigators. All interviews were conducted in a place where the interviewer was sure that there were no third parties listening to the interview. All the young persons sampled were informed that their participation in the study was voluntary, with the possibility of withdrawing from the study without any negative ramifications.

Powered by Editorial Manager® and ProduXion Manager® from Aries Systems Corporation

**Format for specific study types**<br><br>**Human Subject Research (involving human participants and/or tissue)**<br>• Give the name of the institutional review board or ethics committee that approved the study<br>• Include the approval number and/or a statement indicating approval of this research<br>• Indicate the form of consent obtained (written/oral) or the reason that consent was not obtained (e.g. the data were analyzed anonymously)<br><br>**Animal Research (involving vertebrate animals, embryos or tissues)**<br>• Provide the name of the Institutional Animal Care and Use Committee (IACUC) or other relevant ethics board that reviewed the study protocol, and indicate whether they approved this research or granted a formal waiver of ethical approval<br>• Include an approval number if one was obtained<br>• If the study involved *non-human primates*, add *additional details* about animal welfare and steps taken to ameliorate suffering<br>• If anesthesia, euthanasia, or any kind of animal sacrifice is part of the study, include briefly which substances and/or methods were applied<br><br>**Field Research**<br><br>Include the following details if this study involves the collection of plant, animal, or other materials from a natural setting:<br>• Field permit number<br>• Name of the institution or relevant body that granted permission	
**Data Availability**<br><br>Authors are required to make all data underlying the findings described fully available, without restriction, and from the time of publication. PLOS allows rare exceptions to address legal and ethical concerns. See the PLOS Data Policy and FAQ for detailed information.	Yes - all data are fully available without restriction

A Data Availability Statement describing where the data can be found is required at submission. Your answers to this question constitute the Data Availability Statement and **will be published in the article**, if accepted.<br><br>**Important:** Stating 'data available on request from the author' is not sufficient. If your data are only available upon request, select 'No' for the first question and explain your exceptional situation in the text box.<br><br>Do the authors confirm that all data underlying the findings described in their manuscript are fully available without restriction?	
**Describe where the data may be found in full sentences. If you are copying our sample text, replace any instances of XXX with the appropriate details.**<br><br>• If the data are **held or will be held in a public repository**, include URLs, accession numbers or DOIs. If this information will only be available after acceptance, indicate this by ticking the box below. For example: *All XXX files are available from the XXX database (accession number(s) XXX, XXX.).*<br>• If the data are all contained **within the manuscript and/or Supporting Information files**, enter the following: *All relevant data are within the manuscript and its Supporting Information files.*<br>• If neither of these applies but you are able to provide **details of access elsewhere**, with or without limitations, please do so. For example:<br><br>*Data cannot be shared publicly because of [XXX]. Data are available from the XXX Institutional Data Access / Ethics Committee (contact via XXX) for researchers who meet the criteria for access to confidential data.*<br><br>*The data underlying the results presented in the study are available from (include the name of the third party*	All relevant data are within the manuscript and its Supporting Information files.

and contact information or URL).<br>• This text is appropriate if the data are owned by a third party and authors do not have permission to share the data.<br><br>* typeset	
Additional data availability information:	

Manuscript

Click here to access/download;Manuscript;Acceptability of PrEP_Submitted_28th Dec 24.doc

Acceptability of pre-exposure prophylaxis and associated factors among HIV-negative young men in Kagwara fishing community- Serere district: a cross-sectional study

Short Title: PrEP acceptability among HIV negative young men.

Authors: Alex Omoding[1,2], Ronald Opito[3**], Paul Oboth[4], Francis Okello[1], Joseph KB Matovu[1,5]

Author affiliations

[1]Department of Community and Public Health, Faculty of Health Sciences, Busitema University, Mbale, Uganda

[2]Department of Health, Uganda Protestant Medical Bureau (UPMB), Kampala, Uganda.

[3]Department of Public Health, School of Health Sciences, Soroti University, Soroti, Uganda.

[4]Department of Biochemistry and Molecular Biology, Faculty of Health Sciences, Busitema University, Mbale, Uganda

[5]Department of Disease Control and Environmental Health, Makerere University School of Public Health, Kampala, Uganda

**Corresponding author: dopito@sun.ac.ug.

Key words: HIV Pre-Exposure Prophylaxis, acceptability, ~~factors,~~ young men, fishing community

Abstract

Background: Despite the potential efficacy of Pre-Exposure Prophylaxis (PrEP) in reducing HIV risk among at-risk populations, PrEP acceptability remains strikingly varied across

populations. We assessed PrEP acceptability and associated factors among at-risk HIV-negative young men in Kagwara fishing community, Serere district.

Methods: A cross-sectional quantitative study design was used. Data were collected among 409 at-risk HIV-negative young men aged 15-24 years living in Kagwara fishing community, between August and October 2023. Quantitative data were collected on socio-demographic characteristics, sexual-risk behaviors, knowledge, attitudes, and practices regarding PrEP acceptability. PrEP acceptability was defined as the proportion of young men accepting to use PrEP out of those interviewed, based on six constructs adopted from acceptability framework. Data analyzed using Stata version 15.0 statistical software. Summary statistics computed and presented as tables, frequencies and proportions. Bivariate analysis was conducted using penalized logistic regression to identify independent factors associated with PrEP acceptability. All factors that had $p<0.10$ at the bivariate analysis and suspected confounders were entered into the final logistic regression model. All factors with $p<0.05$ were significantly associated with the primary outcome.

Results: Of 409 respondents, average age was 21.8(SD=1.9) years. Majority, (97.8%, n=393) had unprotected penetrative sex while 84.6% (n=346) did not know HIV status of their partners. PrEP acceptability was high at 93.6% (n=383) as majority of the participants accepted to use PrEP based on the six constructs of acceptability. At multivariable level, the factors associated with PrEP acceptability were; perceived risk of getting HIV infection, adjusted odds ratio (aOR)(95%CI)=4.23(1.05, 17.04), knowing the partner's HIV status, aOR (95%CI) = 0.25 (0.07, 0.88), feeling embarrassed to ask for PrEP from the facility, aOR (95%CI) = 0.12 (0.04, 0.39), and having concern of stigma associated with use of PrEP, aOR = 0.13 (95% CI, 0.04-0.41).

Conclusion: We found a high level of PrEP acceptability among at-risk HIV-negative young men in Kagwara fishing community. Improving access to PrEP services among at-risk young men in the fishing communities may increase PrEP uptake in this population and across similar settings.

Introduction

Despite the availability of recognized and recommended Pre-Exposure Prophylaxis (PrEP) for HIV prevention among at-risk populations, new HIV infections have persisted and are highest among adolescents and young people [1]. Globally, 1.3 million people were newly infected with HIV in 2022 and infections occurring among young people aged 15-24 years contributed 20% [2]. In Uganda, an average of 52,000 people become infected with HIV every year, with 36% of these new infections occurring among adolescents and young people [3]. The HIV prevalence in Uganda has a geographical heterogeneity with fishing communities listed among those with high prevalence [4]. The HIV prevalence among young fisher-folks is at least twice as high as among young people in the general population [3]. This could be attributed to the multiple co-occurring transitions during adolescence such as increased autonomy, decreased adult supervision, identity formation, peer influence, substance abuse (alcohol and other illicit drugs), emotional and social transition, potentially leading to early sexual debut and health risk behaviors that may lead to HIV infections [5–7]. In addition, fishing communities have increased susceptibility to HIV due to complex interacting factors like high mobility, poor access to healthcare including HIV prevention and care services, low perception of HIV risk, and poor access to HIV information [8]. Also, fishing communities are known for having sexually active persons who engage in risky

sexual intercourse and high populations who engage in transactional sex [9–11], to which some young people get inclined [12,13].

Uptake of PrEP among at-risk people in the fishing communities remains largely undocumented, yet its success as an intervention greatly depends on its acceptability [14,15]. According to studies across several populations, PrEP acceptability has varied from as low as 1% among the young people in South Africa [16] to as high as above 90% among the female sex workers in Zambia [17]. Most PrEP implementation efforts have focused primarily on reaching at HIV high-risk populations, especially female sex workers, men who have sex with men, HIV discordant couples, adolescent girls and young women because they account for the majority of new HIV infections [17–21]. Subsequent studies on PrEP use in settings providing standard of care have also focused on the same populations leaving out young men who are also sexually active and are sexual counterparts of women who are at high-risk of HIV infection. Therefore, this study aimed at assessing acceptability of pre-exposure prophylaxis and associated factors among at-risk HIV-negative young men in Kagwara fishing community- Serere district to provide supportive evidence towards provision and scale up of PrEP use.

Methods and Materials

Study design This study adopted a cross-sectional design, and a random sampling technique was used in which a representative population of at-risk HIV-negative young men in the fishing community of Kagwara was studied at one instance each. The design was chosen because it incorporated the use of questionnaire as the data capture tool, with the implication that it was possible to collect quantifiable data that was required to answer the research questions. In

addition, collection of not only descriptive data that could be analyzed but also the possibility of analyzing relationships between any descriptive variables of interest [22,23]. In other words, the design allowed for the analysis of all possible factors associated with PrEP acceptability among the at-risk HIV negative young men.

Study setting

This study was conducted in Kagwara fishing community; Serere district. Kagwara fishing community was chosen as the study site because just like other fishing communities; HIV incidence is high [24] and it is being targeted for PrEP provision yet the targeted persons therein have largely demonstrated hesitance towards HIV prevention methods for reasons not yet established by the ministry of health or its partners. There were certainly several associated factors to that behavior that needed to be assessed whether PrEP would be accepted before it is introduced. Kagwara fishing community is one of the fishing communities in Lake Kyoga found in Eastern part of Uganda. It is being served by Kagwara HC III, one of the health facilities in Kadungulu sub-county.

Study population

The study population was young men who were sexually active, having concurrent multiple (two or more) sexual partners, young men reporting no or inconsistent condom use with women of unknown HIV status or HIV positive women and young men with known HIV- negative test result. Young men who were medically compromised to provide informed consent and young men who did not identify with their birth gender were excluded from the study.

Study variables

Dependent variable

The dependent variable (primary outcome) for this study was acceptability of HIV PrEP which was defined as proportion of at-risk HIV-negative young men that would express intention to use PrEP for HIV prevention purpose if made available to them at no cost. PrEP acceptability was derived as a composite variable from six out of seven theoretical constructs of acceptability framework [TFA] [25]. These included: affective attitude, burden, perceived effectiveness, intervention coherence, self-efficacy and opportunity cost. Although the acceptability framework includes seven theoretical constructs, we used six constructs because the seventh construct (Ethicality) had an ambiguous question which was not straight-forward and therefore confused the participants. Each theoretical construct had one question with five responses on a liked scale. The five responses were collapsed into two (2), that is, positive, scored one (1) and negative, scored as zero (0). This helped in the final computations, and it avoided scores cancelling the result. The sum of the six responses per participant was computed and ranged from zero (0) to six (6). Participants with scores of 4-6 were considered as having accepted PrEP while those with scores of 0-3 were considered as having not accepted PrEP.

Independent variables: The independent variables in this study were factors associated with PrEP acceptability that included the sociodemographic characteristics such as age, marital status, education level, religion, behavioral characteristics such as having multiple sexual partners, knowing the partners' HIV status, use of drugs of abuse and other medical related conditions such as having ever been diagnosed with sexually transmitted infection (STI).

Data collection procedures and methods

The study participants were recruited and data collected between August-October 2023. An introductory letter was obtained from Busitema University and approval sought from the district health officer of Serere district. The approved introductory letter was then used for introducing research team to the management of the fishing community. The governor is the overall leader of the fishing community who works in conjunction with recognized government arms like local councils, police and local government officials like parish chief and sub-county chief. The young men were then accessed through these leaders of the fishing community. Their age eligibility (15-24 years) to participate in the study was verified using the documents obtained at the landing site from the governor but also others who mentioned verbally their years were considered and more so those who corroborated their years of birth with special events that had happened in the specified period of 15-24 years (1994-2008) and this helped in calculating the actual years.

On daily basis working with community leaders' young men found doing activities of the day would be approached and the purpose of the visit explained. Those who accepted to participate in the study would randomly be selected and the subsequent procedures would then be conducted that included further screening for study eligibility, offering HIV counselling and testing and those who turned negative would then be interviewed following research questionnaire that had been configured in KOBO tool.

Data analysis

Data were extracted from the KOBO tool and exported to STATA (version 15.0) statistical software for analysis. Data were analyzed at 3 different levels. Descriptive statistics such as mean, frequencies, standard deviations and range were done and presented as tables, frequencies and proportions. Bivariate analysis was conducted using penalized logistic regression to compare

independent predictors of PrEP acceptability with the outcome of interest. The final multivariable analysis involved using penalized logistic regression to determine the factors associated with acceptability of PrEP among this population. These were reported as adjusted odds ratios (aOR) and factors whose confidence intervals of the OR did not include a null (1.0) were considered statistically significant.

Results

Socio-demographic characteristics of the respondents

Table 1 shows the socio-demographic characteristics of the study population. Overall, 409 respondents participated in the study. The average age of the respondents was 21.8 years (SD: ±1.9). Most (87.8%, n=359) of the respondents were aged 20-24 years and 12.2% (n= 50) were aged 15-19 years. About 61.9% (n=253) of the respondents had primary education as their highest level of education reached while 21.5% (n=88) had attended secondary education and 1% (n=4) had tertiary education and 15.6% (n= 64) of the respondents had no formal education. Slightly above three quarters (79%, n=323) were fishermen and more than half (68.4%, n=280) were married.

Table 1. Socio-demographic characteristics of the respondents

**Variables**	**Frequency ~~(N=409)~~**	**Proportion (%)**
**Mean age/years**	21.8(SD=1.9)	
**Age ~~group (Complete years)~~**		
15-19 ~~years~~	50	12.2%
20-24 ~~years~~	359	87.8%
**Education Level**		

None	64	15.6%
Primary	253	61.9%
Secondary	88	21.5%
Tertiary	4	1.0%
**Occupation**		
Business	6	1.5%
Fishing	323	79.0%
Peasant	45	11.0%
Salaried	2	0.5%
Self-employed	2	0.5%
Student	31	7.6%
**Marital Status**		
Single	129	31.6%
Married	280	68.4%

Behavioral characteristics of the respondents

Table 2 shows the behavioral characteristics of the respondents. More than half (59.2% n=242) of the respondents had multiple sexual partners. Most (97.8%, n=393) of the respondents had un-protected sexual intercourse within six months of the study. About 84.6% (n=346) did not know their partner(s) HIV status. More than half (52.6%, n=215) used drugs of abuse and 61.1% (n=250) of the respondents had never heard about PrEP. All (100%, n= 409) of the respondents were aware of the availability of HIV services at the health facilities. Most (91.9%, n=376) of the respondents would not feel embarrassed to ask for PrEP from health facilities if made available freely. More than three quarters (78.5%, n=321) of the respondents would take PrEP because of perceived risk of HIV. About 63.1% (n=258) of the respondents would take PrEP because of having multiple sexual partners. Three quarters (76.5% n=313) of the respondents would take

PrEP because of having sex without condoms. About 78.5% (n=321) of the respondents would take PrEP because of having sexual partners of unknown HIV status and most (95.6%, n= 391) of the respondents would take PrEP because of history of sexually transmitted disease.

Table 2. Behavioral characteristics of the respondents

Variable	Frequency	Percentage
**Number of Sexual Partners**		
One	167	40.8%
More than one	242	59.2%
**Was penetrative sex protected ~~(N=404)~~**		
No	393	97.8%
Yes	13	3.2%
**Know partner HIV status**		
No	346	84.6%
Yes	63	15.4%
**Used addictive drugs in the last 6 months**		
No	215	52.6%
Yes	194	47.4%
**Ever heard about PrEP**		
No	250	61.1%
Yes	159	38.9%
**HIV services offered with other services at the facility**		
No	0	0.0%
Yes	409	100.0%
**Feeling embarrassed to ask for PrEP at facility**		
No	376	91.9%
Yes	33	8.1%
**Perceived risk of HIV infection motivates me to use PrEP**		
No	88	21.5%

Yes	321	78.5%
**Having multiple sexual partners motivates me to use PrEP**		
Yes	258	63.1%
No	151	36.9%
**Having sex without condoms motivates me to use PrEP**		
Yes	313	76.5%
No	96	23.5%
**Having sexual partners of unknow HIV status motivates me to use PrEP**		
Yes	321	78.5%
No	88	21.5%
**History of sexually transmitted disease**		
Yes	391	95.6%
No	18	4.4%

Concerns about HIV PrEP

Table 3 shows concerns about HIV PrEP highlighted by the at-risk HIV negative young men in Kagwara fishing community. Almost three quarters (72.9%, n=298) of the respondents had concerns about PrEP. More than half (55.7%, n=166) of the respondents with concerns pointed out less effectiveness of below one hundred percent. About 58.1% (n=173) of respondents mentioned side effects. More than quarter (29.5%, n=88) of the respondents pointed out costs associated with PrEP. About 39.9% (n=119) of the respondents with concerns raised a concern of pill burden. Almost a third (31.9%, n=95) of the respondents pointed out stigma. About 12.4% (n= 37) of respondents were not comfortable of using PrEP while at the same time using condoms and a few 1.3% (n=4) of the respondents with concern pointed out the health workers negative attitude as some of the concerns about HIV PrEP.

Table3. Concerns about HIV PrEP

Variable	Frequency	Percentage
**Having any PrEP concerns**		
No	111	27.1%
Yes	298	72.9%
**Effectiveness of less than 100% (N=298)**		
No	132	44.3%
Yes	166	55.7%
**Side effects concern (N=298)**		
No	125	41.9%
Yes	173	58.1%
**Cost concerns (N=298)**		
No	210	70.5%
Yes	88	29.5%
**Pill burden concern (N=298)**		
No	179	60.1%
Yes	119	39.9%
**Stigma concern (N=298)**		
No	203	68.1%
Yes	95	31.9%
**Still must use condom concern (N=298)**		
No	261	87.6%
Yes	37	12.4%
**Health workers negative receptivity concern (N=298)**		
No	294	98.7%
Yes	4	1.3%

Key: The numbers in this table are varying because the first question was asked to all respondents, and the subsequent questions were asked only respondents who had concerns regarding PrEP

PrEP acceptability based on the constructs of theoretical framework of acceptability

Table 4 shows PrEP Acceptability based on 6 out of 7 constructs of the theoretical framework of acceptability (TFA). Overall, 93.6% (n=383) of the at-risk HIV negative young men ~~in Kagwara fishing community~~ accepted PrEP for HIV prevention purpose. Most (91.4%, n=373) of the respondents had positive attitude towards PrEP and were comfortable. Three quarters (76.5%, n=313) of the respondents felt there was no burden associated with PrEP and strongly mentioned that it will take little or no effort to use PrEP. About 82.4% (n=337) of the respondents had a good perception of PrEP effectiveness and pointed that PrEP is likely to reduce their chance of getting infected with HIV. Most (92.9%, n=380) of the respondents agreed that it was clear to them how using PrEP reduces chances of getting infected with HIV (intervention coherence). About 89.5% (n=366) of the respondents were confident of being able to use PrEP (self-efficacy) and most 90.9% (n=372) of the respondents disagreed that using PrEP would interfere with their other priorities (opportunity costs).

Table 4. PrEP Acceptability based on the Theoretical Framework of Acceptability

Construct	Question Asked	Response (Score)	Frequency	Proportions (%)
Affective Attitude	How comfortable do you feel to use PrEP?	Comfortable (1)	374	91.4
		Uncomfortable (0)	35	8.6
Burden	How much effort will it take to use PrEP?	Little/No effort (1)	313	76.5
		A lot of effort (0)	96	23.5
Perceived Effectiveness	PrEP is likely to reduce my chance of getting infected with HIV	Agree (1)	337	82.4
		Disagree (0)	72	17.6
Intervention coherence	It is clear to me how using PrEP reduces my chances of getting infected with HIV	Agree (1)	380	92.9
		Disagree (0)	29	7.1
Self-Efficacy	How confident do you feel that you can do what is required to use PrEP	Confident (1)	366	89.5
		Unconfident (0)	43	10.5
Opportunity Cost	Using PrEP will interfere with my other priorities	Agree (0)	37	9.1
		Disagree (1)	372	90.9
PrEP Acceptability	Total of the 6 scores is 4 or more	Yes (Scores≥4)	383	93.6
		No (Scores<4)	26	6.4

Factors associated with PrEP acceptability

Table 5 shows the factors associated with PrEP acceptability among the at-risk HIV negative young men in Kagwara fishing community. At a multivariable level, young men who perceived

HIV risk had four times higher odds of accepting PrEP compared to their counterparts who never perceived HIV risk, aOR(95%CI) =4.23(1.05-17.04). Young men who knew their partners' HIV status had lower odds of accepting PrEP compared to those who did not know their partners HIV status, aOR (95%CI) =0.25(0.25 (0.07-0.88). Those who felt embarrassed to ask for PrEP from the facility had lower odds of accepting PrEP, aOR(95%CI) =0.12(0.04-0.39), and young men who were concerned about stigma of using PrEP had lower odds of accepting PrEP, aOR (95%CI) = 0.13(0.04- 0.41).

Table 5. Factors associated with PrEP acceptability among at-risk, HIV-negative men in Kagwara fishing community.

**Characteristic**	**Willing to accept PrEP**		**Odds Ratio (OR)**		**P-Value**
	**Yes (n, %) 383 (93.6%)**	**No (n, %) 26 (6.4%)**	**Crude OR (95%CI)**	**Adjusted OR (95%CI)**	
**Age group**					
15-19 years	39(78.0)	11(22.0)	1.00	1.00	
20-24 years	344(95.8)	15(4.2)	**6.47(2.82-14.85)**	2.98(0.80-11.13)	0.104
**Highest level of education level attained**					
Primary	295(93.1)	22(6.9)	1.00	1.00	
Post-primary	88(95.7)	4(4.3)	1.49(0.53-4.24)	1.00(0.21-4.85)	0.995
**Occupation**					
Fisherman	307(95.1)	16(4.9)	1.00	1.00	
Non-fisherman	76(88.4)	10(11.6)	**0.39(0.17-0.88)**	1.96(0.47-8.14)	0.351
**Marital status**					
Single	111(86.1)	18(13.9)	1.00	1.00	
Married	272(97.1)	8(2.9)	**5.32(2.29-12.34)**	2.64(0.82-8.49)	0.103
**Number of sexual partners**					
One	149(89.2)	18(10.8)	1.00	1.00	

>One	234(96.7)	8(3.3)	**3.41(1.48-7.89)**	3.01(0.95-9.56)	0.061
**Known partner HIV status**					
No	326(94.2)	20(5.8)	1.00	1.00	
Yes	57(90.5)	6(9.5)	0.56(0.22-1.40)	**0.25(0.07-0.88)**	**0.031**
**Used addictive drugs in the last 6 months**					
No	198(92.1)	17(7.9)	1.00	1.00	
Yes	185(95.4)	9(4.6)	1.72(0.76-3.89)	1.30(0.38-4.44)	0.672
**Ever heard about PrEP**					
No	229(91.6)	21(8.4)	1.00	1.00	
Yes	154(96.9)	5(3.1)	**2.63(1.01-6.86)**	1.03 (0.29-3.61)	0.968
**Feeling embarrassed to ask for PrEP at facility.**					
No	364(96.8)	12(3.2)	1.00	1.00	
Yes	19(57.6)	14(42.4)	**0.05(0.02-0.11)**	**0.12(0.04-0.39)**	**0.000**
**Perceived risk of HIV acquisition motivates me to use PrEP.**					
No	81(92.0)	7(8.0)	1.00	1.00	
Yes	302(94.1)	19(5.9)	1.43(0.59-3.43)	**4.23(1.05-17.04)**	**0.042**
**Effectiveness of less than 100%**					
No	121(91.7)	11(8.3)	1.00	1.00	
Yes	153(92.2)	13(7.8)	1.08(0.47-2.45)	0.35(0.10-1.17)	0.088
**Side effects concern**					
No	115(92.0)	10(8.0)	1.00	1.00	
Yes	159(91.9)	14(8.1)	1.00(0.44-2.29)	0.52(0.18-1.51)	0.227
**Stigma concern**					
No	199(98.0)	4(2.0)	1.00	1.00	
Yes	75(79.0)	20(21.0)	**0.08(0.03-0.24)**	**0.13(0.04-0.41)**	**0.001**

Notes: Bold=Significant with P<0.05, All factors were adjusted for each other. P-Values indicated are for aOR.

Discussion

This study assessed HIV PrEP acceptability and associated factors among the at-risk HIV negative young men ~~in Kagwara fishing community- Serere district~~. ~~Almost all (93.6%) of the respondents accepted PrEP for HIV prevention.~~ This higher acceptability of PrEP shows a positive progress in HIV prevention efforts. Previous studies documented low PrEP acceptability among various populations. Among young men PrEP acceptability ranged from 45.45% to 76.80% in Sub-Saharan Africa (SSA) [26] . In Uganda, PrEP acceptability has been equally low in the earlier studies. For instance the study done in the fishing community of Lake Victoria found PrEP acceptability ranging from 14%-24% [27]. Subsequent studies have showed increasing PrEP acceptability among various populations. For example Bashir Ssuna et al reported 8 in 10 fisher folks in Kampala were willing to use PrEP [28]. Likewise, Susan S Witte reported 9 in 10 female sex workers were willing to initiate PrEP in Uganda [29]. This higher acceptance could have resulted from sustained behavior change messages targeting high risk communities such as fisher folks in the fishing communities and the adoption and promotion of PrEP as a key biomedical strategy for HIV prevention by ministry of health of Uganda [30]. The acceptability of PrEP by these groups therefore provides an opportunity for the ministry of health to expand access to PrEP services to reach the communities as one of a combination prevention strategy to reaching HIV epidemic control in Uganda. Ministry of health needs to use multiple approaches to provide PrEP such as peer-led models, drug distribution points, short message reminders for refills, pharmacies and retail drug shops

We found perceived risk of HIV infection as a positive factor associated with increased PrEP acceptability among the at-risk HIV negative young men ~~in Kagwara fishing community.~~ This implies that most of the young men engage in sexual relationships without knowing their

partners' HIV status. The perception here can be attributed to limited HIV testing in most of the communities in Uganda and young men practice unsafe sex which results in increased spread of HIV. This finding agrees with other studies that found a relationship between perceived risk of HIV and PrEP acceptability [31–33]. It is important to make PrEP services available along with HIV-tests and build capacity of the young men in performing HIV self-tests. In addition, various HIV testing modalities that are user friendly should be readily available like HIV self-testing at both facility and community.

Our study found knowing partners' HIV status as one of the factors associated with reduced PrEP acceptability. This could be attributed to mutual trust developed by the partners. This finding is contrary with those of Ssuna et al. who did their study in Uganda and found knowledge of HIV status being associated with high PrEP use [34]. They reported that study participants who tested for HIV within the past 6 months were more likely to use PrEP. This requires packaging of HIV prevention messages in focused manner, educating young men about HIV window period and use of multiple HIV prevention methods like circumcision to cater for those that will not accept PrEP.

Our study also revealed that feeling embarrassed to ask for PrEP from the health facilities was another factor that reduced PrEP acceptability among the young men in Kagwara fishing community. This can be attributed to inferiority and high illiteracy levels common in the fishing communities in Uganda. This finding is in line with the study conducted among transgender women in U.S.A that identified being embarrassed with health care system as one of the factors affecting PrEP acceptability [35]. There is a need to train and orient health workers on customer care to handle such young men, introduce and sustain youth friendly health services at both

health facilities and communities with focus in places where young men always have leisure activities.

In addition, our study found concern of stigma associated with PrEP use as another factor that reduced PrEP acceptability among the young men in Kagwara fishing community. Stigma has been associated with reduction of health care acceptability particularly in chronic illness like HIV. This finding has been also reported by study conducted among transgender women in U.S.A [36]. there is a need to break this barrier through continued sensitization of the young men to embrace the available HIV prevention methods.

Study limitations and strengths

Our study had some limitations that included limited previous research studies on acceptability of PrEP by at-risk HIV-negative young men and this might have affected the theoretical foundation of our research, and this was managed by using studies on PrEP acceptability done in the other populations. We did not provide physical PrEP to study participants but only reported their presumed acceptability and in case they are to be given real PrEP pills, actual acceptability might reduce during implementation. The recruitment of only men affected overall acceptability level for the population and limited external validity and extrapolation of findings to the general population. Some participants might have had prior knowledge or education on PrEP and this might have insinuated some responses.

Despite these limitations, our study had strengths: This was among the few studies conducted among the at-risk HIV negative young men in the fishing community which is one of the unique populations prone to HIV, the knowledge gained from the present study may aid in lowering HIV infections among the at-risk HIV negative young men, findings can help to inform efforts to

increase health promotion and continued sanitization to encourage young men to accept PrEP. Finally, findings in this study can serve as foundation for future researchers, policy makers and health professionals.

Conclusion

Our study revealed that HIV PrEP is acceptable by at-risk HIV negative young men ~~in Kagwara fishing community~~. The factors associated with HIV PrEP acceptability were perceived risk of HIV, knowing partner(s) HIV status, feeling embarrassed to ask for PrEP from health facilities and concern of stigma associated with PrEP use. These findings indicate the need to promote HIV PrEP among at-risk HIV negative young men in the fishing communities with intensified health promotion messages. There is a need to continue promoting use of multiple HIV prevention approaches and create awareness on the people eligible for PrEP.

It is important to identify barriers to PrEP acceptability among this population using a qualitative approach. This will help in designing person centered approach to PrEP delivery among young men in the fishing community.

List of abbreviations

**AGYW**	Adolescent Girls and Young Women
**AIDS**	Acquired Immunodeficiency Syndrome
**AVERT**	Aid Virus Education and Research Trust
**AYFRHS**	Adolescent and Youth Friendly Reproductive Health Care Services
**CDC**	Centers for Disease Control and Prevention
**DHIS2**	District Health Information System Software version 2

**HIV**	Human Immunodeficiency Virus
**HMIS**	Health Management Information System
**MSM**	Men who have sex with Men
**MSMW**	Men who have Sex with Men and Women
**NRTIs**	Nucleotide Reverse Transcriptase Inhibitors
**PrEP**	Pre-exposure prophylaxis
**PWID**	People Who Inject Drugs
**PWUD**	People Who Use Drugs
**STD**	Sexually Transmitted Disease(s)
**UN**	United Nations
**UNAIDS**	United Nations Joint Program on HIV
**UNFPA**	United Nations Fund for Population Activities
**UNICEF**	United Nations International Children's Emergency Fund
**WHO**	World Health Organization

Declarations

Ethical approval and consent to participate

Ethical approval to conduct the study was obtained from Busitema University Research and Ethics Committee (REC), Approval number **BUFHS-2023-61**. Administrative clearance was obtained from the district health office, Serere district and the local government representative office in whose jurisdiction Kagwara landing site was situated. Given that the study was assessing behavior related to HIV prevention among high-risk HIV- negative persons, this certainly needed to uphold several ethics when conducting the study. Each participant at his own

discretion provided an informed consent (written) as was indicated on the consent form, while parental consent was obtained for all the minors prior to their participation in the study. Confidentiality was maintained by ensuring that individual patient-level data obtained was de-identified, encrypted, and passworded to ensure access by only an authorized team of investigators. All interviews were conducted in a place where the interviewer was sure that there were no third parties listening to the interview. All the young persons sampled were informed that their participation in the study was voluntary, with the possibility of withdrawing from the study without any negative ramifications.

Consent for publication. Not applicable

Availability of data and materials. All relevant data are within the manuscript and its Supporting Information files.

Competing interests. The authors declare that they have no competing interests.

Funding: The authors did not receive any funding for this study.

Authors' contributions:

AO, PO and JKBM conceptualized and developed the proposal. AO and JKBM supervised data collection. RO and FO conducted data curation and formal analysis. RO and AO drafted the manuscript. PO and JKBM reviewed the final manuscript. All authors read and approved the final manuscript.

Acknowledgements

The authors would like to acknowledge the contribution of all the staff of the Department of Public and Community Health, Faculty of Health Sciences, Busitema University for their support during the design and implementation of the study. We would also like to appreciate the technical support from Dr.Ogwal Daniel, the district health officer Serere district who provided administrative clearance and oversaw data collection process in the landing site.

REFERENCES

1. Khalifa A, Stover J, Mahy M, Idele P, Porth T, Lwamba C. Demographic change and HIV epidemic projections to 2050 for adolescents and young people aged 15-24. Glob Health Action [Internet]. 2019;12(1). Available from: https://doi.org/10.1080/16549716.2019.1662685
2. UNAIDS, UNAIDS Global AIDS update. The path that ends AIDS. 2023;80–2. Available from: http://www.wipo.int/amc/en/mediation/rules
3. Ministry of Health Uganda. Uganda Population - Based Hiv Impact assessment. 2019;0–252.
4. Uganda AIDS Commussion. “Ending the HIV and AIDS epidemic: Communies at the forefront.” 2020;(August). Available from: http://www.uac.go.ug
5. UNAIDS. UNAIDS Data 2018. 2018;1–376.
6. Cicchetti D, Rogosch FA. A developmental psychopathology perspective on adolescence. J Consult Clin Psychol. 2002;70(1):6–20.
7. Schulenberg J, Maggs JL, Hurrelmann K. Health risks and developmental transitions during adolescence [Internet]. Health risks and developmental transitions during

adolescence. 1997. p. xviii, 580. Available from: https://books.google.co.za/books?hl=en&lr=&id=9_l9mjWblb4C&oi=fnd&pg=PA23&dq=cultural-historical+context+&ots=FTIMPP7xfy&sig=M8qm3H2xlQ-WReGF2L06HnskIXs#v=onepage&q=cultural-historical context&f=false%0Ahttps://ovidsp.ovid.com/ovidweb.cgi?T=JS&CSC=Y&NEWS=

8. Kiwanuka N, Ssetaala A, Nalutaaya A, Mpendo J, Wambuzi M, Nanvubya A, et al. High incidence of HIV-1 infection in a general population of fishing communities around Lake Victoria, Uganda. Vol. 9, PLoS ONE. 2014.

9. Sileo KM, Bogart LM, Wagner GJ, Musoke W, Naigino R, Mukasa B, et al. HIV fatalism and engagement in transactional sex among Ugandan fisherfolk living with HIV. Sahara J. 2019;16(1):1–9.

10. Bradford K, Katikiro RE. Fighting the tides: A review of gender and fisheries in Tanzania. Vol. 216, Fisheries Research. 2019. p. 79–88.

11. Moore E V., Nambi R, Isabirye D, Nakyanjo N, Nalugoda F, Santelli JS, et al. When Coffee Collapsed: An Economic History of HIV in Uganda. Med Anthropol Cross Cult Stud Heal Illn. 2022;41(1):49–66.

12. C.H. L, M. O, S. M, R. H, H. A, Kyambadde P. AO - Logie Heather; ORCID: https://orcid.org/0000-0001-6410-4616 AO - Okumu, Moses; ORCID: https://orcid.org/0000-0003-2555-3077 AO - Mwima, Simon; ORCID: https://orcid.org/0000-0003-2745-2026 AO - Kyambadde, Peter; ORCID: https://orcid CHO https://orcid. org/000.-0002-8035-433X AO-A. Gender, transactional sex, and HIV prevention cascade engagement among urban refugee and displaced adolescents and youth in Kampala, Uganda [Internet]. Vol. 33, AIDS Care - Psychological and Socio-Medical

Aspects of AIDS/HIV. 2021. p. 897–903. Available from: https://www.tandf.co.uk/journals/titles/09540121.asp%0Ahttp://ovidsp.ovid.com/ovidweb.cgi?T=JS&PAGE=reference&D=emed22&NEWS=N&AN=2007645150

13. Kyegombe N, Meiksin R, Namakula S, Mulindwa J, Muhumuza R, Wamoyi J, et al. Community perspectives on the extent to which transactional sex is viewed as sexual exploitation in Central Uganda. BMC Int Health Hum Rights. 2020;20(1):1–16.

14. Scandurra C, Bochicchio V, Dolce P, Valerio P, Muzii B, Maldonato NM. Why people were less compliant with public health regulations during the second wave of the Covid-19 outbreak: The role of trust in governmental organizations, future anxiety, fatigue, and Covid-19 risk perception. Curr Psychol. 2023;42(9):7403–13.

15. Al-Hasan A, Khuntia J, Yim D. Does Seeing What Others Do Through Social Media Influence Vaccine Uptake and Help in the Herd Immunity Through Vaccination? A Cross-Sectional Analysis. Front Public Heal. 2021;9(November):1–13.

16. Ajayi AI, Mudefi E, Yusuf MS, Adeniyi OV, Rala N, Goon D Ter. Low awareness and use of pre-exposure prophylaxis among adolescents and young adults in high HIV and sexual violence prevalence settings. Vol. 98, Medicine (United States). 2019.

17. Ortblad KF, Chanda MM, Musoke DK, Ngabirano T, Mwale M, Nakitende A, et al. Acceptability of HIV self-testing to support pre-exposure prophylaxis among female sex workers in Uganda and Zambia: Results from two randomized controlled trials. Vol. 18, BMC Infectious Diseases. 2018.

18. Harawa N, Bempong G, Fields S, Leibowitz AA, Lee D, Meyer P, et al. Engaging Black Men Who Have Sex with Men (BMSM) in Los Angeles in HIV Pre-Exposure Prophylaxis (PrEP). 2016; Available from: http://chipts.ucla.edu/wp-content/uploads/2016/02/White-

Paper-Black-MSM-and-PrEP.pdf

19. Jared,M.B, Ph.D., Ph.D. DD, Patrick Ndase, M.B., Ch.B. MPH, Nelly R. Mugo, M.B., Ch.B., M.P.H. JDC, Jonathan Wangisi MB, Ch.B., M.Sc., Jordan W. Tappero, M.D. MPH, et al. Antiretroviral Prophylaxis for HIV-1 Prevention among Heterosexual Men and Women. Bone [Internet]. 2011;23(1):1–7. Available from: https://www.ncbi.nlm.nih.gov/pmc/articles/PMC3624763/pdf/nihms412728.pdf

20. Baeten JM, Heffron R, Kidoguchi L, Mugo NR, Katabira E, Bukusi EA, et al. Integrated Delivery of Antiretroviral Treatment and Pre-exposure Prophylaxis to HIV-1–Serodiscordant Couples: A Prospective Implementation Study in Kenya and Uganda. Vol. 13, PLoS Medicine. 2016.

21. Atkins K, Rucinski K, Mudavanhu M, Holmes L, Mutunga L, Kaufman MR, et al. Sexual Relationship Types, Partner HIV Self-Testing, and Pre-Exposure Prophylaxis Among South African Adolescent Girls and Young Women: A Latent Class Analysis. J Acquir Immune Defic Syndr. 2021;86(4):413–21.

22. Munnangi S, Boktor SW. Epidemiology Of Study Design - PubMed [Internet]. Stat Pearls Publishing. 2022. p. 1–3. Available from: https://pubmed.ncbi.nlm.nih.gov/29262004/

23. Setia MS. Methodology series module 3: Cross-sectional studies. Vol. 61, Indian Journal of Dermatology. 2016. p. 261–4.

24. Musumari PM, Techasrivichien T, Srithanaviboonchai K, Wanyenze RK, Matovu JKB, Poudyal H, et al. HIV epidemic in fishing communities in Uganda: A scoping review. Vol. 16, PLoS ONE. 2021.

25. Sekhon M, Cartwright M, Francis JJ. Acceptability of healthcare interventions: An overview of reviews and development of a theoretical framework. BMC Health Serv Res

[Internet]. 2017;17(1):1–13. Available from: http://dx.doi.org/10.1186/s12913-017-2031-8

26. Kakande A, Ssemata AS, Muhumuza R, Atujuna M, Abaasa A, Ndekezi D, et al. Preferences for oral PrEP dosing among adolescent boys and young men in three sub-Saharan African countries. Vol. 18, PLoS ONE. 2023.

27. Kuteesa MO, Quaife M, Biraro S, Katumba KR, Seeley J, Kamali A, et al. Acceptability and Predictors of Uptake of Anti-retroviral Pre-exposure Prophylaxis (PrEP) Among Fishing Communities in Uganda: A Cross-Sectional Discrete Choice Experiment Survey. Vol. 23, AIDS and Behavior. 2019. p. 2674–86.

28. Ssuna B, Katahoire A, Armstrong-Hough M, Kalibbala D, Kalyango JN, Kiweewa FM. Factors associated with willingness to use oral pre-exposure prophylaxis (PrEP) in a fisher-folk community in peri-urban Kampala, Uganda. Vol. 22, BMC Public Health. 2022.

29. Witte SS, Filippone P, Ssewamala FM, Nabunya P, Bahar OS, Mayo-Wilson LJ, et al. PrEP acceptability and initiation among women engaged in sex work in Uganda: Implications for HIV prevention. Vol. 44, eClinicalMedicine. 2022.

30. Ministry of Health. Technical Guidance on Pre-Exposure Prophylaxis (PrEP) for Persons at Substantial Risk of HIV Infection in Uganda Ministry of Health. 2022;1–58. Available from: www.health.go.ug

31. Walters SM, Kral AH, Simpson KA, Wenger L, Bluthenthal RN. HIV Pre-Exposure Prophylaxis Prevention Awareness, Willingness, and Perceived Barriers among People Who Inject Drugs in Los Angeles and San Francisco, CA, 2016–2018. Vol. 55, Substance Use and Misuse. 2020. p. 2409–19.

32. N. P de C, C.C.P. M, R.C.F. C, D.J.D. A, C.A. M de P. HIV pre-exposure prophylaxis

(PrEP) awareness and acceptability among trans women: a review [Internet]. Vol. 31, AIDS Care - Psychological and Socio-Medical Aspects of AIDS/HIV. 2019. p. 1234–40. Available from: http://www.embase.com/search/results?subaction=viewrecord&from=export&id=L627555383%0Ahttp://dx.doi.org/10.1080/09540121.2019.1612014

33. Sila J, Larsen AM, Kinuthia J, Owiti G, Abuna F, Kohler PK, et al. High Awareness, Yet Low Uptake, of Pre-Exposure Prophylaxis among Adolescent Girls and Young Women within Family Planning Clinics in Kenya. Vol. 34, AIDS Patient Care and STDs. 2020. p. 336–43.

34. Ssuna B, Katahoire A, Armstrong-Hough M, Kalibbala D, Kalyango JN, Kiweewa FM. Factors associated with willingness to use oral pre-exposure prophylaxis (PrEP) in a fisher-folk community in peri-urban Kampala, Uganda. BMC Public Health [Internet]. 2022;22(1):1–8. Available from: https://doi.org/10.1186/s12889-022-12859-w

35. Chakrapani V, Shaikh S, Arumugam V, Chawla U, Mehta S. Factors Influencing Willingness to Use Human Immunodeficiency Virus Preexposure Prophylaxis among Transgender Women in India. Vol. 6, Transgender Health. 2021. p. 64–73.

36. Ogunbajo A, Storholm ED, Ober AJ, Bogart LM, Reback CJ, Flynn R, et al. Multilevel Barriers to HIV PrEP Uptake and Adherence Among Black and Hispanic/Latinx Transgender Women in Southern California. Vol. 25, AIDS and Behavior. 2021. p. 2301–15.

Supporting Information

S1: Dataset on Acceptability of PrEP

S2: REC Approval Letter.

Click here to access/download
Supporting Information
REC APPROVAL LETTER.pdf

Click here to access/download
Supporting Information
S1_Dataset_Acceptability of PrEP.xlsx
