# Supplementary material for: Acceptability of pre-exposure prophylaxis and associated factors among HIV-negative young men in Kagwara fishing community-Serere district, Uganda: A cross-sectional study
Source: PLoS One. 2025 Jun 4;20(6):e0317808. doi: 10.1371/journal.pone.0317808 (PMC12136446; doi:10.1371/journal.pone.0317808)
Supplement: Attachment — Submitted filename: Point-by-Point_response_Acceptability of PrEP.pdf [file pone.0317808.s006.pdf]

April 15th, 2025

The Editor,
PLos One Journal

Re: Point-by-point response to reviewers' comments on our manuscript "Acceptability of HIV pre-exposure prophylaxis and associated factors among at-risk HIV-negative young men in Kagwara fishing community- Serere district: a cross-sectional study"

We are excited to receive positive review feedback on our manuscript titled, "**Acceptability of HIV pre-exposure prophylaxis (PrEP) and associated factors among at-risk HIV-negative young men in Kagwara fishing community- Serere district: a cross-sectional study**" which we had submitted to your prestigious journal, PLoS One.

Below is the point-by-point response to the reviewers' comments, marked in red, in addition to the tracked changes in the main documents.

Reviewer #1: Congratulations on working on a topic of public importance locally and internationally. You need to work on the issue that I have highlighted in the PDF manuscript file which I have attached.

We have addressed all the issues highlighted in the PDF as recommended.

-The study objectives do not line up well with the title of the study.

We have agreed to focus on the acceptability of PrEP and its associated factors rather than combining it with KAP. As such the title remained unchanged.

-The methodology also lacks good clarity and somehow is disorganized. Key issues like how calculation of PrEP acceptability was done is not clear.

The methodology has been reorganized. A description of how PrEP acceptability was calculated has been included under the subsection of measures of variables. In brief, PrEP acceptability was measured as a composite variable from six constructs of theoretical framework of acceptability [TFA].

Reviewer #2: Thank you for the opportunity to review this manuscript. Young men are truly underserved and less is known about their needs concerning HIV prevention. Below are some comments:

Title: Include Uganda. This has been included in the title.

Key words: Review to HIV; pre-exposure prophylaxis; delete factors, include Uganda. This has been adjusted as recommended.

Abstract: Line 22. Which PrEP is being referred to? Oral, Injectable? TDF/FTC, please clarify, also in the main background. We were referring to oral PrEP and we have made clarifications in the abstract and the introduction sections as recommended.

Line 25 include Uganda. This has been included.

Line 28: delete quantitative. Done

Line 31.. Include statistics were. Done

Line 38- include SD in full. Done

Line 45: delete having concern. Wouldn't that be low acceptability/use of PrEP?

Response. The language in the results section has been re-organized.

Conclusion, what strategies can be recommended for teh challenges identified?

Response. The Ministry of health needs to use multiple approaches to provide PrEP such as peer-led models, drug distribution points, short message reminders for refills, pharmacies and retail drug shops.

Introduction

Line 54, update to UNSIDS 2024 data and reference,

This had been updated accordingly.

Line 59, include statistics. The statistics have been included.

Line 64, the sentence starting with in addition is not clear whether it refers to communities or people This was refereeing to people in the fishing communities

Line 67, delete sexually active. Those who use PrEP must be those that are sexually active. Deleted as guided.

Line 68, delete high populations who.. Deleted

Throughout the manuscripts probably refer to high risk young men instead of including HIV. This is noted and adjusted accordingly.

Methods

Line 86- review to this was a cross-sectional study. Done

Re-organize the whole section under sub sections: study design, site, population, data collection, analysis, etc. Please include a section for participant recruitment to cater for information on how the young men were identified. This has been done and the section on participant recruitment included.

Line 95 and following, most of the information should be under participant recruitment. The study setting would include details about Serere and what activities take place in fishing communities, and details about health care. This section has been revised as recommended.

Line 106 provide details of inclusion and exclusion criteria. Exclusion should not necessarily be the opposite of the inclusion criteria. The details have been provided.

Line 146, which activities? Activities such as boat cleaning and preparation, fish smoking/drying, selling fish or those on their activities of leisure such as playing cards, watching football or betting at the landing site

Line 150- tested instead of turned?.. those who tested negative were interviewed using.... What is the KOBO tool? Any reference? The web page has been included as a reference.

Line 158, which variables were fit to be included in the model? How was that determined?

Variables included in the final model were sociodemographic and behavioral characteristics, determined using a their plausibility and a P-Value cut off of 0.10 at bivariate level.

Line 162, what was the level of significance? The level of significance was set at 5%.

For all the results in text, the percentages are actual, using the word about is misleading.

This is noted and adjusted accordingly.

Results

Line 167 SD in full. This has been written in full.

Line 168 and following, there would be a focus on one side of the proportion instead of listing all. The section has been reorganized as recommended.

Table 1: SD should have the Plus/minus symbol. Occupation, clarify on what business, peasant salaries stand for.

The Plus/Minus sign has been included. Occupation has been reorganized as fisherman or non-fisher man.

Line 176 and following, refer to illicit drug use instead of drugs of abuse/ addiction, etc,

Thank you, this has been adjusted accordingly.

Line 225, bivariate findings are not presented. The bivariate findings are presented and summarized in table 5, with significant factors at bivariate level bolded. These were not written in text to reduce the work and focus on the significant factors in the final multivariate level.

Line 237, table footer, what does adjusted for each other mean? This has been removed.

Line 277- review thoughts around window period. Revised accordingly.

Line 280- review factor was associated with no acceptability...Revised accordingly.

Line 284- need to provide youth friendly services? The sentence has been restructured.

Line 308-309 is a recommendation. The piece has been taken to the recommendation section.

Line 319- among people eligible? The sentence has been corrected.

Line 328, any approval from UNCST? This was a student-led study (for Master of Public Health from Busitema University) and as such ethical clearance from an approved REC was sufficient for the conduct the study as per the UNCST guidelines.

Lines 330-332 are redundant. Removed.

Was assent obtained? Did the study recruit emancipated minors? Was parental consent obtained? Both assent and parental consent were obtained for the minors who were enrolled in the study.

Lines 337, not easy to confirm that the place had no one else available. Please review. This has been reviewed.

Line 338-340 is also redundant. Removed.

Reference: Please include access data for all web-based references.

Thes have been included.

Generally, review grammar and sentence construction throughout the manuscript.

This has been reviewed and revised accordingly.

Regards,

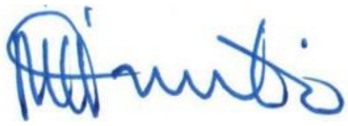

Dr. Ronald Opito
Corresponding Author
